# What are the reasons for unfinished nursing care as perceived by hospitalized patients? Findings from a qualitative study

**DOI:** 10.1111/hex.13652

**Published:** 2022-11-22

**Authors:** Stefania Chiappinotto, Alberto Coppe, Alvisa Palese

**Affiliations:** ^1^ Medical and Surgical Department University of Tor Vergata Roma Italy; ^2^ Health Care Professionals Service AULSS 2 Marca Trevigiana Treviso Italy; ^3^ Department of Medical Sciences University of Udine Udine Italy

**Keywords:** causes, patient engagement, qualitative research, reasons, unfinished nursing care

## Abstract

**Introduction:**

Unfinished nursing care (UNC), as the care required by patients that delayed or not delivered, has been investigated mainly from the perspective of nurses, while little is still known from the side of patients. Some studies have involved patients to measure which elements of care are mostly unfinished (e.g., mouth care), whereas a few studies have investigated the reasons for UNC as perceived by them. Their involvement in understanding the reasons for UNC is crucial to advance the knowledge and co‐develop possible strategies to prevent or minimize UNC.

**Methods:**

This is a descriptive qualitative study performed according to COnsolidated criteria for REporting Qualitative research guidelines in 2022. A purposeful sample of Italian hospitalized patients in two medical and two surgical units was involved. A face‐to‐face semistructured interview was used to merge reasons for UNC. Qualitative content analysis was conducted to merge subthemes and themes as factors leading to UNC according to the experience of patients.

**Results:**

A total of 23 patients (12 surgical and 11 medical) were involved (12/23 male) with an age average of 66.2 years, educated mainly at secondary school, and with previous hospitalizations (20/23), and dependent on nursing care in daily activities (14/23). Reasons for UNC have been identified at four levels: (1) ‘New health‐care system priorities’ and ‘Pre‐existing frailty of health‐care facilities’ were reasons identified at the health‐care system level; (2) ‘Lack of resources attributed to wards’, ‘Ineffective ward organization’ and ‘Leadership’ were identified at the unit level; (3) ‘Nurses' attitudes and behaviour’ were reported at the nurses' level and (4) ‘Increased nursing care expectations’ were pinpointed at the patient level.

**Conclusion:**

Patients can be involved in identifying UNC, but also in recognizing the underlying reasons. Engaging them in such investigations might broaden our understanding of the phenomenon and the possibility of identifying strategies to minimize and prevent UNC.

**Patient or Public Contribution:**

Patients from four hospital units (two medical and two surgical) were involved in face‐to‐face interviews to merge the reasons perceived by them as triggering UNC. All factors (as themes and subthemes) have derived from their words, thus enhancing the evidence available from the side of the patients.

## 1 INTRODUCTION

Unfinished nursing care (UNC)^1^ has been widely investigated as a concept,^2^ in terms of its antecedents^3^ and consequences (e.g., Wieczorek‐Wojcik et al.^4^). This phenomenon has been documented in the literature under different terms, such as ‘tasks left undone’,^5^ ‘implicit rationing of nursing care’^6^ and ‘missed nursing care’.^7^ Over time, these different frameworks have highlighted various aspects of the phenomenon,^8^ but emerging evidence agrees on the fact that UNC is experienced by nurses and patients as an important issue for the quality of care,^9^, ^10^, ^11^ as it refers to nursing care that is required but completely omitted or delayed. However, this phenomenon has been investigated mainly from the nurses' angle, whereas little is still known from the patients' side.

The first study involving patients was performed by Kalisch et al.^12^ using a qualitative method to explore the extent and type of missed nursing care as experienced by them. The authors developed a list of questions centred on activities that patients should have received, asking them if nurses had been able to complete them. Findings have highlighted that some activities were fully reportable as missed by patients (e.g., mouth care and bathing), others were partially reportable (e.g., hand washing and assessing vital signs) while others were not (e.g., nursing care process). Therefore, patients were not able to report all UNC elements, but their perceptions were considered important to gain the global picture of the phenomenon as perceived by them; however, the reasons for missed nursing care were not investigated.

Two years later, another study^13^ assessed the missed nursing care phenomenon using the MISSCARE Survey‐patient, by developing it from that already validated among nurses.^14^ This quantitative study was aimed at investigating the amount and type of missed nursing care as perceived by patients and the patient‐reported outcomes. Patients referred to basic care, communication and time to respond to needs as the most omitted or postponed care. However, the ‘Reason for Missed Nursing Care’ section of the MISSCARE Survey for nurses, which assessed the perceived causes, was not administered to patients because during the pilot testing the most frequent answer to various items was ‘I do not know’.

Three further studies quantify patients' perceptions of missed nursing care under the Kalisch framework. First, Dabney and Kalisch^15^ performed a study aimed at investigating the relationship between nursing staffing and the patients' report on missed nursing care, using the MISSCARE Survey‐patient. They found a correlation between the total nursing staff hours of care per patient‐day, registered nurse hours per patient‐day, registered nurse skills mix and the occurrence of missed care; however, no exploration was conducted on the reasons for missed care.

By using the MISSCARE Survey‐patient to investigate patients' perceptions, Cho et al.^16^ explored the mediating effects of missed nursing care as reported by patients on the relationship between nursing staffing and patients' experiences. An association between better staffing adequacy, less missed care and better patient experiences emerged, indicating that patient perception of missed care mediated the relationship between staffing adequacy and their own experiences.

However, only Moreno‐Monsiváis et al.^17^ investigated nurses' and patients' perceptions using the MISSCARE Nursing Survey, providing some modifications to the tool to collect patients' points of view regarding causes. In the attempt to expand the knowledge available, the section concerning the reasons perceived was retained and adapted by asking to patients ‘Why do you think nurses do not “always” provide some aspects of care?’. Patients reported the lack of staff, the insufficient experience of the staff, the lack of organization and teamwork, the lack of staff communication from one shift to another and the attitude of staff members as reasons for missed nursing care.

In addition to studies based on the Kalish framework, Orique et al.^18^ administered the Hospital Consumer Assessment of Healthcare Providers and Systems (HCAHPS) tool,^19^ a national standardized instrument developed by the Centers for Medicare & Medicaid Services and the Agency for Healthcare Research and Quality for assessing patients' perception of hospital care. Researchers used some items that were useful for investigating elements of missed nursing care in an acute care setting, but again in this case no questions were raised about the reasons for missed care.

Therefore, according to our best knowledge, the only qualitative study to have investigated UNC with a missed nursing care framework as perceived by patients was the one by Kalisch et al.^12^ Some other quantitative studies have collected patients' perceptions about this phenomenon, but underlying reasons as perceived by them were little investigated. Second, available data have been collected mainly under the missed nursing care conceptualization, while in recent years a more comprehensive framework capable of including all different concepts in this field by establishing the UNC umbrella concept.^20^ Moreover, involving patients in investigating the reasons for UNC might contribute to expanding the knowledge available by including a wider perspective. The concept of patient engagement has also assumed a fundamental role in detecting issues and in promoting the quality of care.^21^, ^22^ In a world where citizens require a health system to be transparent, open and responsive, patients' engagement has become imperative and an effective strategy for understanding their experiences and for promoting alliances with them with a view to achieving better care.^23^ Evidence has documented that engaging patient increases their safety,^24^, ^25^ their satisfaction with care^26^ and last but not least, their healthcare outcomes.^27^ Moreover, deepening an understanding of patients' experiences has been seen as the first step towards patients' engagement.^23^ Following this perspective of involvement and engagement, the purpose of this study was to explore the reasons for UNC as perceived by patients, thus going beyond the point of view of nurses as mainly perspective included to date.

## 2 METHODS

### 2.1 Study design

This is a descriptive qualitative study,^28^ performed in 2022 and reported here according to the COnsolidated criteria for REporting Qualitative research guidelines^29^ (Supporting Information: Table 1). Moreover, the study was designed under the UNC framework^1^ to ensure (a) inclusiveness of all different conceptual traditions in the field and (b) an updated approach is given that tools and investigations in recent years have been conducted under the UNC framework.^30^

### 2.2 Setting

A large healthcare trust of the Veneto Region Public Health Care Service, comprised of seven hospitals and four accredited facilities, equipped with a total of 2390 beds serving 880,000 citizens in 2021, of which 23% were >65 years old, was approached. Among those available, one large hospital was identified (35,000 admissions/year^31^), and within these, two medicals (66 beds each) and two surgical units (52 beds/each) were considered for the study.

### 2.3 Participants

A purposeful sample of patients^32^ with rich knowledge about, or experience with, the phenomenon of interest was chosen. Specifically, patients were included if they (a) were adults (>18 years); (b) had been hospitalized for more than 48 h; (c) was on discharge or with a planned discharge, and thus were not unstable or in their acute phase; (d) were able to participate in an interview and (e) were willing to participate in the study. Therefore, those patients not meeting the inclusion criteria were excluded.

The recruitment process was conducted daily from the start of the study: a researcher (S. C., see authors), who was an advanced educated nurse (PhD student and research fellow), and was not involved in the care of patients, consulted the nurse responsible for the nursing care or the nurse manager to decide on the patient to approach. The recruitment ended when data saturation was achieved,^33^ as judged independently by two researchers (S. C. and A. P.; see authors), when dominant themes were perceived as completed and no others emerged from the interviews. None of the identified patients refused to participate.

### 2.4 Data collection

According to the only study available that collected patients' perceptions about UNC with a qualitative approach,^12^ and considering the most recent studies investigating reasons for UNC as perceived by nurses,^34^, ^35^, ^36^ a semistructured interview was designed. The interview was composed of the following open‐ended questions:

(1) Demographic data and the perceived degree of dependence in the activity of daily living (e.g., I am independent; I need help in some daily activities [eating, hygiene]);
(2) A recall of a particular UNC episode; and
(3) A full description of the underlying reasons according to the perceptions/experience of the patient (Table 1). The interview guide was pilot tested on the first four participants. No changes were necessary.

**Table 1:** Interview guide

Interview guide
*Introductory section*
Researcher self‐presentation
Presentation of the study aim and of data collection procedures
Acquisition of written consent for the interview and the audio‐recording
*First section*
Demographic data, perceived degree of dependence in activities of daily living and previous hospitalization
Age
Gender
Education
Working profile
Functional dependence (yes/no)
Previous hospitalizations (yes/no)
*Second section*
Unfinished nursing care and reasons
Recall of a particular episode of UNC
Narration of the perceived reasons triggering the episode narrated according to personal experience
Additional elements considered relevant in the context of UNC experienced

Abbreviation: UNC, unfinished nursing care.

The interviews were scheduled for between April and June 2022 and were conducted face‐to‐face on a day and at a time preferred by each patient. No relationship with participants was established before the commencement of the study. They were informed only about the working position of the researcher and the aims of the study, which were illustrated in a detailed fashion by the nurse responsible for the patient and then repeated by the researcher at the time of the interview. The interviews, which lasted for between 3 and 22 min, were carried out in a quiet setting, where only the researcher and the participant were present.

### 2.5 Data analysis

A qualitative content analysis^37^ was used to merge subthemes and themes describing reasons for UNC as perceived by patients. Specifically, two researchers (S. C. and A. P.; see authors) performed the analysis by (a) transcribing the interviews; (b) reading and rereading the transcriptions, and also by contextually selecting the units of meaning (i.e., a word or sentence that holds a specific meaning in the context of perceived UNC reasons); (c) identifying subthemes: each researcher identified subthemes (i.e., an abstraction of the units of meaning labelled with a code) independently, as derived from the data. Then, a consensus was reached between researchers regarding the subthemes that emerged; after having reached the consensus, researchers proceeded by (d) categorizing the subthemes. As in the previous step, each researcher identified the themes by grouping subthemes independently; the agreement was reached by consensus through multiple meetings. An example of the coding tree is reported in Supporting Information: Table S2.

The data analysis was performed manually, without using any software. The coding process was initiated immediately, after three interviews, and then continued to assess the saturation when reached^33^ as judged when no new subthemes emerged. The concurrent analysis of the data as immediately performed after the interview, allows to limit the number of participants, given that being involved in understanding the reasons for the poor quality of care may burden patients, especially when they are still hospitalized.

### 2.6 Rigour and truthfulness

Several strategies have been enacted to ensure rigour and truthfulness.^38^ First, the understandability and feasibility of the questions included in the interview were ensured throughout the pilot test. Second, the credibility of the findings and the data dependability were ensured by extracting quotations to provide concrete examples of reasons from the words of participants, and by reporting the number casual assigned to each participant (e.g., P6, Participant number 6) to ensure anonymity. Third, the end of the interviews was decided according to the data saturation as assessed by two researchers, who evaluated in an independent fashion and then compared subthemes that emerged. Fourth, to prevent the influence of preconceptions, the coding process was conducted by two researchers independently by using anonymized data and then agreeing on findings; moreover, the quality of the process was ensured by involving researchers who were experts in qualitative methods and in interviews. Furthermore, transferability was promoted by describing the settings involved and the participants' main profiles.

### 2.7 Ethical issues

The Ethical Committee approved the study protocol (16th December 2021, prot. n. 234258/2021; Amendment 31st March 2022). Participants were informed of the study aims, and they were free to withdraw from the study at any time without any consequence. They were also ensured that the interview would not be shared with nurses responsible for their care and all data would be anonymized. At the end of the explanation, they were asked to sign the consent form where they also agreed to be audio‐recorded.

The researchers anonymized the narratives before the data analysis, assigning a casual number to each participant interviewed; moreover, the wards were anonymized, and thus their official names were changed to prevent them from being recognized. Quotations were also identified with the number of participants.

## 3 RESULTS

### 3.1 Participants

As reported in Table 2, a total of 23 patients were involved, most of them male (12/23) with a mean age of 66.2 years (standard deviation 14; range 40–92). Most of the patients reported a secondary school education (14/23), followed by an elementary school (6/23) and some were educated at the university level (3/23). Most participants were retired (14/23). Moreover, 20 out of 23 reported previous hospital experiences in different wards, 12 had been cared for in surgical units and 11 in medical units. The majority (14/23) perceived the need for help due to functional dependence on activities of daily living.

**Table 2:** Participants' characteristics

ID	Gender	Age	Education	Ward	Work position	Functional dependency	Previous hospitalization
1	F	74	Elementary school	Medical α	Retired	No	Yes
2	F	89	Elementary school	Medical α	Retired	Yes	Yes
3	M	60	Secondary school	Medical α	Designer and production manager	No	Yes
4	F	56	Secondary school	Medical α	Embroiderer	No	Yes
5	F	65	Elementary school	Medical β	Retired	Yes	Yes
6	M	69	Master Degree	Medical β	Retired	No	Yes
7	M	60	Secondary school	Medical β	Retired	No	Yes
8	M	79	Secondary school	Medical β	Retired	Yes	Yes
9	F	57	Bachelor	Surgical α	Freelancer	No	No
10	M	51	Secondary school	Surgical α	Taxi company manager	Yes	Yes
11	F	92	Elementary school	Surgical α	Retired	Yes	Yes
12	M	57	Secondary school	Surgical α	Retired	No	Yes
13	F	77	Master	Surgical β	Retired	Yes	Yes
14	M	40	Secondary school	Surgical β	Owner of a company	Yes	Yes
15	F	75	Elementary school	Surgical β	Retired	Yes	Yes
16	M	44	Secondary school	Surgical β	Truck driver	Yes	No
17	M	71	Secondary school	Medical α	Retired	Yes	Yes
18	F	59	Secondary school	Medical α	Housewife	Yes	Yes
19	F	87	Elementary school	Medical α	Retired	Yes	Yes
20	M	47	Secondary school	Surgical α	Digital video entrepreneur	No	Yes
21	F	67	Secondary school	Surgical α	Farmer	Yes	No
22	M	76	Secondary school	Surgical α	Retired	No	Yes
23	M	71	Secondary school	Surgical α	Retired	Yes	Yes

Abbreviations: F, female; M, male.

### 3.2 UNC reasons

As summarized in Table 3, the reasons for UNC have been identified at four levels, namely at the healthcare system, at the unit, at the nurses and at the patient levels, including seven subthemes. The ‘New healthcare system priorities’ and the ‘Pre‐existing frailty of healthcare facilities’ were reasons identified at the healthcare system level, and the ‘Lack of resources attributed to wards’, the ‘Ineffective ward organization’ and ‘Leadership’ were identified at the unit levels; the ‘Nurses' attitudes’ and ‘Behavior’ were reported at the nurses' level while the ‘Increased nursing care expectations’ at the patient level. Moreover, as reported in Table 3, some reasons were reported only by patients hospitalized in medical or surgical units.

**Table 3:** Levels, themes and subthemes

Level	Themes	Subthemes	Medical ward	Surgical ward
Healthcare System	New healthcare system priorities	Cost restraints	a	a
		Dramatic changes due to the COVID‐19 pandemic		a
	Pre‐existing frailty of healthcare facilities	Unsuitable environment layout	a	a
		Old technologies	a	a
		Discrepancies in resource allocation across wards		a
Unit	Lack of resources attributed to wards	Staff shortages	a	a
		High patient‐to‐nurse ratio	a	a
	Ineffective ward organization	General vocation of the ward		a
		Poor nursing care delivery design	a	a
		Poor shift design		
		Lack of staff during the day, nights and weekends Excessive length of shifts Lack of care continuity between shifts	a a	a
		Overlapping activities	a	a
		High frequency of interruptions		a
		Limited capacity to react to unpredictable events		
		Admissions Emergencies		a
			a	
	Ineffective ward leaders	Inadequate nurse manager leadership		a
Nurses	Nurses' competences and attitudes	Lack of delegation skills	a	
		Lack of empathic competences	a	
		Lack of responsibility	a	a
		Low motivation	a	a
		Living in a hurry	a	a
		Expressed fatigue	a	a
Patients	Increased nursing care needs and care expectations	Worse clinical conditions	a	a
		Increased ADL dependence	a	a
		Demanding patients		a

Abbreviation: ADL, activities of daily living.

^a^ Reported by patients hospitalized in this ward.

### 3.3 UNC reasons at the healthcare system level

Two main themes have emerged at this level. Patients reported that UNC is due to the ‘New health‐care system priorities’, where the quality of care has started not to be identified among the top priorities in recent years. In other words, patients reported UNC as an inevitable consequence of the ‘cost restraints’ applied in the last few decades to the entire system, reducing progressively the funding, and affecting the number of staff employed in hospitals:

You cannot always cut on the number of personnel … Health care is based on the quality and the quantity of the personnel. (P6)

Participants have also underlined the effects of the ‘dramatic changes due to the COVID‐19 pandemic’, where new priorities were established marking a turning point in nursing care delivery, further reducing resources in some units, especially in medical and surgical ones, to devote them to COVID‐19 wards, thereby increasing the risk of care omissions.

Also, now for the COVID situation, I have seen … I've been going inside out of hospitals for 10 years and I've seen a great negative change. (P10)

Patients reported that the emerging priorities greatly affect the ‘Pre‐existing frailty of health‐care facilities’; among these, the ‘unsuitable environment layout’, due to old‐fashioned hospital buildings, has been reported as affecting nurses' timely responses to the needs of patients, due to the time required to reach each patient's room or the nurse station, thereby increasing the risk of delays in care.

Yes, because sometimes they are closer, sometimes they are further away. (P21)

Patients have also highlighted the role of ‘old technologies’ as a factor influencing the occurrence of UNC, where nurses are still using papers and pencils and dedicating a lot of time to filling in them, thus staying away from patients:

…the lack of the more advanced technologies. (P9)

Moreover, participants also perceived ‘discrepancies in resource allocation across wards’ as a reason for UNC, where human and material resource allocation across settings is unbalanced, leading to an excess of some resources and a paucity in others:

Therefore, I saw discrepancy in resources within the same department. (P9)

### 3.4 UNC reasons at the unit level

Three main themes emerged at the unit level, namely a ‘Lack of resources attributed to wards’, the ‘Ineffective ward organization’ and the ‘Ineffective ward leaders’, all of which led to UNC according to the patients' perceptions.

Among the first of these, participants stressed ‘staff shortages’, as the number of all staff, ranging from nursing aides to nurses, was below the minimum standard required to manage all care:

Few [staff], few, very few… (P11)

In the specific context of nurses, participants also reported a ‘high patient‐to‐nurse ratio’, as identified by the nurses themselves:

So, nurses themselves say: ‘We are undersized, it would take more professionals’. (P3)

Alongside the resources allocated at the unit level, patients also reported the role of an ‘ineffective ward organization’. The ‘general vocation of the ward’ was considered a reason for UNC, given that according to previous patients' experience, specialized wards were able to ensure greater attention to individual needs, delivering more complete care:

…[nurses] provide a better care in a specialized ward than a general medicine ward. (P9)

Moreover, the ‘poor nursing care delivery design’ was found to be a reason for UNC, due to the chaotic environment and the nonoptimal care processes, where participants relieved nurses from being responsible for their omissions:

…the service is badly organized; it is not the fault of the nurses. The organization of the service is terrible. (P6)

The poor organization has also been reported as being complicated by the ‘poor shift design’: the lack of nurses during the day resulting in high workloads and the need to postpone some activities was perceived as an issue preventing the completion of care, especially in the mornings.

During the day they take a little longer; in the evening they are faster. (P20)

Also, during the night and at the weekend, patients reported being cared for by a lower number of staff than expected for managing all needs. The same duration of shifts was reported as being a reason for UNC because it affected the performance of nurses:

…with shifts too long. They [nurses] could do broken shifts…. (P6)

On the other hand, patients reported a lack of continuity of care between shifts as increasing omissions, as nurses have been considered unable to share the main data about patients, leaving out needs perceived by them as important:

…those who were there have left and those who have arrived have just arrived. (P6)

In the attempt to cope with the high workloads, patients often witnessed nurses ‘overlapping activities’ to accelerate the process of care in the desire to ensure all the nursing care required. However, performing several activities at the same time has been reported as a source of delays or omissions:

If she sees a call, she is doing a job and she must finish for other patients, by walking she answers the first patient who has called and then she comes later. (P14)

In the same vein the ‘high frequency of interruptions’ because of patients' calls (P21) thus disrupting the planned activities, has been reported as increasing the number of possible omissions and the capacity to be on time in satisfying multiple needs.

The frailty of the units is further increased by the number of newly admitted patients and emergencies, limiting the nurses' capacity to respond to the needs of patients already present in the unit and in a stable condition, resulting in ‘limited capacity to react to unpredictable events’:

Well, she [nurse] was a little bit late, because maybe a lot of people are admitted here. (P11)

They [nurses] say there are other emergencies and I need to wait for them. (P18)

Above all is the ‘ineffective ward leader’ of the unit, as expressed by patients in his/her capacity to negotiate resources, allocate them properly in the shifts, implement appropriate models of care delivery and support the staff:

…It depends on the ward manager nurse, the head of the ward. That is, these kinds of responsibilities never depend on the last person, you must go up in the hierarchy. (P13)

### 3.5 UNC reasons at the nurses' level

Patients reported some factors also at the nurses' level, specifically highlighting the role of their ‘Competences and attitudes’ as possible reasons for UNC. First, participants referred to a ‘lack of delegation skills’ in some tasks, and thus a higher risk of omitting some relevant nursing activities when workloads increased in intensity:

The nursing aide … the nursing aide can't touch the medicines. Why can't they? (P6)

The ‘lack of empathic competencies’ has also been underlined as triggering omissions in communication, in the understanding of needs, and in responding to them in a timely manner according to the patients' priorities. In addition, patients also reported the perceived ‘lack of responsibility’ and ‘low motivation’ as leading to UNC:

Because it is so convenient for them [nurses] not to do all things. (P19)

…in recent years they [nurses] are all listless. (P17)

On the other hand, patients reported that nurses are always ‘living in a hurry’, thus preventing any contact or interruption by patients to express their needs; sometimes being in a hurry has been reported as the consequence of the excessive workloads, at other times as a question of habit/attitude.

This nurse went away immediately, not even time to finish speaking. Here, when you are still talking and the nurse is already at the door, that is…. (P6)

Moreover, nurses' ‘fatigue’, as explicitly expressed, or as interpreted by patients according to some manifested behaviour as a reaction to the high workloads and the chaotic environment, has been identified as leading to UNC:

Yes, because they are exhausted. (P2)

### 3.6 UNC reasons at the patients' level

Participants have recognized the role of the ‘Increased nursing care needs and care expectations’ in receiving the care required; therefore, while that was sufficient or adequate in the past, today it is never enough because of the ‘worse clinical conditions’ of patients and their ‘increased dependence in daily activities’, determined by co‐morbidities, older age, complex treatments (e.g., medications) and frailty:

And well, of course, when they [nurses] see that you are more stable, they put you a little further back, because there is someone who needs them more. (P12)

I can only say that for the first five days that I could not move, they [nurses] ran here. (P12)

The explosion of nursing care needs presents nurses with a daily challenge in deciding the priorities with the same resources provided to the units years ago. In addition, they must face highly ‘demanding patients’ due to their increased expectations regarding nursing care, rising nurses' workloads and the risk of UNC:

Then I don't know if maybe some periods are different for patients too, maybe at a certain time they are more demanding. (P20)

## 4 DISCUSSION

To the best of our knowledge, this is the first qualitative study investigating the reasons for UNC, as perceived by patients. Adults and older individuals were involved, without applying strict inclusion criteria, resulting in participants educated at different levels, and with different working positions, from active to retired, nearly all with previous hospital experiences and in need of help with basic care. According to the main profile of participants, while gender bias^39^ has been prevented by balancing the genders, the previous hospitalization of patients and their need for basic nursing care suggest that they based their perceptions regarding UNC on their direct experience: patients with poorer health status—as those involved in this study—have been documented as experiencing more UNC.^40^

### 4.1 Methodological discussion

Previous studies in the field have documented that patients are able to recognize and report aspects of UNC mainly regarding basic care, communication and timeliness (e.g., timely help in going to the bathroom).^40^ However, studies investigating their perceptions by using available tools have deleted the questionnaire section regarding the perceived reasons, mainly because ‘Do not know’ was the dominant patients' answer to the items in the pilot surveys.^17^ We undertook the challenge to investigate the reasons for UNC because of the following considerations:

(1) Patients' perceptions reflect a valuable point of view in fully understanding healthcare issues as measured by healthcare professionals.^23^
(2) In the field of patient complaints, the contributory factors leading to problems in care have been neglected, thus focusing their involvement instead on the underlying reasons or causing factors.^41^
(3) Having evidence on perceived reasons for UNC among patients might help to inform them regarding the actual causes thus preventing violence and aggressiveness towards nurses when they are not able to ensure the care required.^42^

However, our study suggests that patients have some difficulties indicating and detecting the reasons for UNC: the interviews were very short in duration, thus indicating that participants were having difficulty in identifying the reasons for the phenomenon. Moreover, some of their perceptions seem to be experienced directly (e.g., overlapping activities), whereas others seem to be experienced indirectly (e.g., a large number of admissions), as reported by (a) the same nurses (e.g., nurse shortages, lack of nurses at the weekend, emergencies) while they try to excuse themselves for the UNC; (b) other patients (e.g., ‘there is a patient with bad clinical conditions’) or (c) by external sources (e.g., newspapers, television), where the information reported may acquire a meaning while hospitalized (e.g., cost restraints). Also in the field of UNC, the perceptions have been differentiated into visible (or fully reportable, or areas of nursing care patients were able to report on), partially and not reportable by patients, which refers to areas of nursing care that patients were unable to report on.^12^ Future studies are recommended to investigate the sources of patients' perceptions, to understand how they develop their understanding regarding the reasons for UNC. Moreover, with the increasing evidence in the field, tools measuring UNC among patients might be completed with the list of possible reasons that emerged in our study.

Some differences have emerged in the UNC factors between medical and surgical wards with some perceived only by patients cared for in medical units (e.g., ‘lack of delegation skills’) and others by those admitted to surgical wards (e.g., ‘inadequate nurse manager leadership’). Studies investigating nurses' perceptions have also reported evidence of some differences.^3^ However, more research is recommended to accumulate evidence in this field to inform different interventions to minimize UNC according to the underlying reasons.

### 4.2 Findings discussion

At the overall level, reasons for UNC have emerged at the healthcare system, unit, nurse and at patient level; previous studies investigating the perceptions of nurses^34^, ^35^, ^36^ have identified the reasons at the system, unit, nurse manager, clinical nurse and patient levels, thus suggesting that patients are able to identify the reasons for UNC at all levels, mirroring the perceptions of nurses.

UNC is affected by several factors, where the upper system, namely the health‐care service priorities, resources, emergencies and values, has been underlined as affecting the care delivered at the bedside.^43^ Patients perceived the relevance of the upper system for the care received daily, suggesting that the long‐term disinvestment in the public health sector, further threatened by the COVID‐19 storm, has reduced the capacity to provide the care required by medical and surgical patients. Apart from the threats to the basic principles of the public healthcare system that are underlined by our participants as compromised (e.g., discrepancies in resource allocation affecting equity), findings suggest that public involvement in setting the priorities, in allocating the resources and in giving feedback on the care ultimately delivered should be core values of policymakers.^44^

At the unit level, patients reported most of the reasons for UNC: hospitalized patients seem to gain an overall picture of factors, underling the importance of resources, models of care delivery and the relevance of the nurse manager leadership. Several reasons reported have already been documented from the side of nurses both in conceptual and empirical evidence, thus confirming the multiple unit factors involved in leading to UNC^3^, ^36^ (e.g., Kalisch & Williams).^14^ However, some have emerged as new from the side of patients, namely: the general vocation of the ward and the lack of care continuity between shifts. All factors that emerged as subthemes seem to be influenced by each other in a sort of domino effect, where the implementation of single interventions to prevent UNC may affect only in part the occurrence of the phenomenon, thus requiring more complex interventions capable of targeting different structural and process elements at the unit level. Moreover, while some factors seem to be modifiable (e.g., poor design of shifts), others are directly connected with the decisions undertaken at the upper level (e.g., the number of resources devoted to nursing care). Furthermore, patients highlighted two main factors worthy of consideration for their ethical implications: the generalist vocation of the units has been reported as a source of UNC, and this should be further investigated and discussed given that most patients are admitted to general wards and they perceive themselves as being at increased risk of UNC compared to those admitted to specialized units; on the other hand, those patients that remain stable during their in‐hospital stay are more at risk of their needs being neglected given that emergencies and newly admitted patients are considered priorities. Equity as well as strategies to prevent any form of discrimination are an imperative principle among nurses, suggesting that these findings should be considered carefully to address appropriate strategies.^45^

Patients also reported factors at the nurses' level by referring to their competencies and attitudes: these findings suggest that some factors rely on individuals, and these may vary across shifts and across nurses, modulating the amount of UNC according to the nurses' individual traits. Previous studies have highlighted the role of individual accountability^46^ as well as that of the nurses' habits as a group.^7^ However, our findings suggest some additional factors: (a) that regarding the competencies in delegating activities and in having an effective relationship with patients, both modifiable through undergraduate and postgraduate education; and (b) that concerning the attitudes of being in a hurry and expressing fatigue. Nurses have the right to demonstrate their difficulties in coping with high workloads and challenging environments, but when these attitudes prevent patients' expression of needs, their ethical implications should be discussed.^47^ Moreover, nurses' attitudes may shape the behaviour of newly graduated nurses and students by encouraging them to conform to a particular approach.^48^ Furthermore, nurses should discuss whether these attitudes are effective when directed to patients in promoting awareness of UNC; instead, identifying strategies to report their emotions, fatigue and difficulties to the healthcare trust headquarters and to the general citizenship rather than to those in need at any given moment might be more effective.

Participants reported that some factors appertain to changed patient profiles, as increased needs and complex clinical conditions trigger increased expectations. Also, in a recent systematic literature review,^3^ patient profiles have been recognized as a factor triggering UNC as perceived by nurses. The fact that the same patients recognized that their needs and expectations have increased means that they are ready to accept all the investments in nursing care that policymakers will provide^49^; nurses should undertake this challenge by educating the future generation to deal with these issues by exercising effective priority setting and by addressing the increased expectations of patients.

### 4.3 Limitations

This study is affected by several limitations. First, no repeated interviews^32^ were performed to explore in greater depth the perceptions of patients during their hospitalization and after discharge—when they might progressively understand the situation and reflect on the entire experience. This decision was undertaken in order not to burden patients. Second, participants member checking^50^ was also not conducted to assess the agreement with the themes and subthemes that emerged as categorized by researchers, given the ample range of reasons reported across patients. Third, only patients capable of participating in an interview were included—missing, therefore, those patients who were not able to answer as well as their close relatives not involved in the process. Relatives might report different perceptions or act as gatekeepers,^51^ whereas patients not able to participate have already been highlighted as being more exposed to UNC,^52^ but the quality of their reporting might be affected by their capacity to understand and interpret the complex situation. Moreover, a few demographic data have been collected and some (e.g., ethnicity, socioeconomic status) were not required to prevent any source of burden on patients. However, future studies should consider extending the data collection to describe in a more detailed fashion the profile of the patients involved.

## 5 CONCLUSIONS

To the best of our knowledge, this is the first study involving patients in identifying the reasons for UNC. Patients reported the causes of UNC at different levels: those close to them (at the unit, at the nurses' and at the patients' level) and those more distant (at the system level). Some UNC reasons reflect those already documented by nurses in the available literature, whereas others appear to be new (e.g., cost restraints, the general vocation of the ward). Moreover, some reasons appear to be perceived directly by patients, while others appear to be mediated by others (other patients, newspapers) and also by nurses when they try to excuse themselves for the omitted or delayed care. However, at the overall level, the rich findings that emerged suggest that patients can be actively involved in identifying the reasons triggering UNC in addition to the elements of nursing care omitted or delayed.

Involving patients in identifying the UNC reasons broadens the understanding of the phenomenon and the possibility of identifying strategies to minimize or prevent it. Furthermore, asking citizens about their perceptions and informing them about the reasons documented, may help them to understand the efforts of nursing staff to ensure the required care, as well as to modulate their expectations in times of resource scarcity, and to act in support of nurses in their attempts to influence policymakers on how to promote the best care.

## AUTHOR CONTRIBUTIONS

This research has been designed and developed by all authors. Stefania Chiappinotto and Alvisa Palese collected the data and carried out the analysis. Results have been interpreted and discussed by Stefania Chiappinotto and Alvisa Palese. Stefania Chiappinotto and Alvisa Palese wrote the first version of the paper. Then, all authors read, commented on, made edits and approved the final manuscript.

## CONFLICT OF INTEREST

The authors declare no conflict of interest.

## Supporting information

Supporting information.

## Data Availability

The data that support the findings of this study are available on reasonable request from the corresponding author. The data are not publicly available due to privacy or ethical restrictions.
