# Supplementary material for: What are the reasons for unfinished nursing care as perceived by hospitalized patients? Findings from a qualitative study
Source: Health Expect. 2022 Nov 22;26(1):256–67. doi: 10.1111/hex.13652 (PMC9854295; doi:10.1111/hex.13652)
Supplement: Supplementary file 1 — Supporting information. [file HEX-26--s001.docx]

**Supplementary Table 1.** COnsolidated criteria for REporting Qualitative research guideline (Tong, Sainsbury & Craig, 2007)

| **No Item** | **Guide questions/description** | **Section** |
| --- | --- | --- |
| ***Domain 1: Research team and reflexivity*** | | |
| *Personal Characteristics* | | |
| 1. Interviewer/facilitator | Which author conducted the interview? | Methods section: participants |
| 2. Credentials | What were the researcher’s credentials? E.g., PhD, MD | Methods section: participants |
| 3. Occupation | What was their occupation at the time of the study? | Methods section: participants |
| 4. Gender | Was the researcher male or female? | Methods section: participants |
| 5. Experience and training | What experience or training did the researcher have? | Methods section: participants |
| *Relationship with participants* | | |
| 6. Relationship established | Was a relationship established prior to study commencement? | Methods section: data collection |
| 7. Participant knowledge of the interviewer | What did the participants know about the researcher? e.g., personal goals, reasons for doing the research | Methods section: data collection |
| 8. Interviewer characteristics | What characteristics were reported about the interviewer/facilitator? e.g., Bias, assumptions, reasons and interests in the research topic | Methods section: data collection |
| ***Domain 2: study design*** | | |
| *Theoretical framework* | | |
| 9. Methodological orientation and Theory | What methodological orientation was stated to underpin the study? e.g., grounded theory, discourse analysis, ethnography, phenomenology, content analysis | Methods section: study design |
| *Participant selection* | | |
| 10. Sampling | How were participants selected? e.g., purposive, convenience, consecutive, snowball | Methods section: participants |
| 11. Method of approach | How were participants approached? e.g., face-to-face, telephone, mail, email | Methods section: data collection |
| 12. Sample size | How many participants were in the study? | Results section: participants |
| 13. Non-participation | How many people refused to participate or dropped out? Reasons? | Methods section: participants |
| *Setting* | | |
| 14. Setting of data collection | Where was the data collected? e.g., home, clinic, workplace | Methods section: setting |
| 15. Presence of non-participants | Was anyone else present besides the participants and researchers? | Methods section: data collection |
| 16. Description of sample | What are the important characteristics of the sample? e.g., demographic data, date | Results section: participants  Table 2 |
| *Data collection* | | |
| 17. Interview guide | Were questions, prompts, guides provided by the authors? Was it pilot tested? | Methods section: data collection  Table 1 |
| 18. Repeat interviews | Were repeat interviews carried out? If yes, how many? | Limitations |
| 19. Audio/visual recording | Did the research use audio or visual recording to collect the data? | Methods section: ethical issues |
| 20. Field notes | Were field notes made during and/or after the interview or focus group? | Methods section: data analysis |
| 21. Duration | What was the duration of the interviews or focus group? | Methods section: data collection |
| 22. Data saturation | Was data saturation discussed? | Methods section: data analysis |
| 23. Transcripts returned | Were transcripts returned to participants for comment and/or correction? | Limitations |
| ***Domain 3: analysis and findings*** | | |
| *Data analysis* | | |
| 24. Number of data coders | How many data coders coded the data? | Methods section: data analysis |
| 25. Description of the coding tree | Did authors provide a description of the coding tree? | Table 3  Supplementary Table 2 |
| 26. Derivation of themes | Were themes identified in advance or derived from the data? | Methods section: data analysis |
| 27. Software | What software, if applicable, was used to manage the data? | Methods section: data analysis |
| 28. Participant checking | Did participants provide feedback on the findings? | Limitations |
| *Reporting* | | |
| 29. Quotations presented | Were participant quotations presented to illustrate the themes / findings? Was each quotation identified? e.g., participant number | Results section: UNC reasons |
| 30. Data and findings consistent | Was there consistency between the data presented and the findings? | Results section: UNC reasons |
| 31. Clarity of major themes | Were major themes clearly presented in the findings? | Results section: UNC reasons |
| 32. Clarity of minor themes | Is there a description of diverse cases or discussion of minor themes? | Results section: UNC reasons |

Abbreviation: UNC, Unfinished Nursing Care

**Supplementary Table 2.** Example of coding tree

| **Quotations** | **Labels** | **Sub-themes** | **Themes** | **Level** |
| --- | --- | --- | --- | --- |
| *You cannot always cut on the number of personnel... The health care is based on the quality and the quantity of the personnel.* (P6) | Founds cut | Cost restrains | Emerging Health Care System priorities | Health Care System |
| *They cut founds a little bit to health care system in my opinion* (P10) |  |  |  |  |
| *Also, now for the COVID situation. I have seen... I’ve been going inside out of hospitals for 10 years and I’ve seen a great negative change.* (P10) | Coronavirus-19 pandemic | Dramatic changes due to the Coronavirus-19 pandemic |  |  |
| *...it was a time of crisis due to pandemic.* (P23) |  |  |  |  |
| *Except that maybe it would also be a smaller ward [referred to another ward].* (P7) | Environment | Unsuitable environment layout | Pre-existing frailty of Health Care facilities |  |
| *Yes, because sometimes they are closer, sometimes they are further away.* (P21) |  |  |  |  |
| *…the lack of the more advanced technologies.* (P9) | Technologies | Old technologies |  |  |
| *Therefore, I saw discrepancy in resources within the same department.* (P9) | Resources allocation | Discrepancies in resources allocation across wards |  |  |

Abbreviation: P, participant; number, the Identify Number casual assigned to each participant to ensure anonymity.
